# A Comparison of Static and Dynamic Functional Connectivities for Identifying Subjects and Biological Sex Using Intrinsic Individual Brain Connectivity

**DOI:** 10.1038/s41598-019-42090-4

**Published:** 2019-04-05

**Authors:** Sreevalsan S. Menon, K. Krishnamurthy

**Affiliations:** Missouri University of Science and Technology, Department of Mechanical and Aerospace Engineering, Rolla, MO 65409 USA

## Abstract

Functional magnetic resonance imaging has revealed correlated activities in brain regions even in the absence of a task. Initial studies assumed this resting-state functional connectivity (FC) to be stationary in nature, but recent studies have modeled these activities as a dynamic network. Dynamic spatiotemporal models better model the brain activities, but are computationally more involved. A comparison of static and dynamic FCs was made to quantitatively study their efficacies in identifying intrinsic individual connectivity patterns using data from the Human Connectome Project. Results show that the intrinsic individual brain connectivity pattern can be used as a ‘fingerprint’ to distinguish among and identify subjects and is more accurately captured with partial correlation and assuming static FC. It was also seen that the intrinsic individual brain connectivity patterns were invariant over a few months. Additionally, biological sex identification was successfully performed using the intrinsic individual connectivity patterns, and group averages of male and female FC matrices. Edge consistency, edge variability and differential power measures were used to identify the major resting-state networks involved in identifying subjects and their sex.

## Introduction

The spontaneous spatiotemporal fluctuations in the brain activity measured using resting-state functional magnetic resonance imaging (rfMRI) time series data have been considered extensively to study functional brain networks since it was first discovered^1^. The correlation of two time series from different regions of the brain or nodes has been used to identify functional connections, and different sets of networks of correlated temporal patterns have led to the identification of resting-state networks (RSNs)^2,3^. Functional connectivity (FC) is denoted as a matrix with the rows and columns representing nodes and each element of the matrix representing the edge strength or functional connection between the corresponding nodes. Although Pearson correlation coefficient is the simplest and most commonly used for defining edge strength, it is not indicative of the direct connection. Partial correlation, on the other hand, is used to estimate the direct connection, which is achieved by regressing out possible indirect connections through other nodes. Using realistic simulated fMRI data, partial correlation was shown to be one of the best methods for network connection detection with high sensitivity compared with various other network models^4^.

A common assumption made in many past studies is that the FC does not change over the data acquisition time period although the brain wanders through a state of connectivities and the FC is non-stationary in nature. A number of recent studies, however, have not made this stationary, or static functional connectivity (sFC), assumption and proposed different methods to study non-stationary changes in connectivity during the rfMRI data acquisition time period, the duration of which is typically from 5 to 15 minutes. The most commonly used approach is that of a windowed analysis^5^ in which the repeated states^6^ are determined using some clustering algorithms^7^. While these methods are more involved and better model the dynamic brain activity, it is not clear how advantageous or what new information can be gleaned by considering the short-term, defined here to be in the order of a few minutes, non-stationarities in the correlation values or dynamic functional connectivity (dFC). Furthermore, it is not clear how the FC change in the medium- to long-term, defined here as months to years, because the temporal evolution of brain connectivity with age has been noted^8–10^. Studies are needed to determine any medium- to long-term changes in the intrinsic individual brain connectivity due to aging or other environmental factors.

Inimitability of functional connectivity profiles and their ability to serve as a ‘fingerprint’ and predict cognitive, behavioral and task performance have been reported in a few studies^11–15^. It has also been reported that edge strengths in some networks are either higher or lower in males, which suggests that the biological sex of a subject may be predicted from the FC matrix^2^. The potential being demonstrated is promising for using intrinsic individual connectivity patterns as ‘neuromarkers’ for studying brain function in health and disease. Further studies are necessary to fully exploit these patterns in this emerging research field^16,17^.

The commonly used sFC and emerging dFC methods were quantitatively compared in this study by evaluating the accuracy of identifying subjects and their sex using their intrinsic individual brain connectivity patterns or ‘fingerprint’. Using publically available data from the Human Connectome Project (HCP) S1200 release, which consists of high quality imaging data from about 1,200 healthy subjects, intra- and inter-subject and male and female group average connectivity patterns were investigated. Although two specific applications of brain connectivity patterns were considered here, this study provides insight into the efficacies of commonly used sFC and dFC analysis methods. Results are also presented that show the intrinsic individual connectivity patterns do not significantly change in the medium term and brain connectivity ‘fingerprinting’ is possible even with data acquired several months apart.

## Methods

### fMRI data

Three sets of preprocessed fMRI data in a grayordinate coordinate system released as a part of the HCP^18^ (https://db.humanconnectome.org/) that have been run through HCP FIX-ICA denoising to remove the effects of structured artefacts were used in this study. In the HCP, data was acquired for about 1,200 young adults (ages 22–35) from families with twins and non-twins using a 3T imaging scanner at Washington University in St. Louis. The fMRI data was acquired with multiband echo-planar imaging at a temporal resolution (TR) of 0.72 s per volume and 2-mm isotropic voxels. A subset of about 100 same-sex twin pairs was studied using a 7T imaging scanner at the University of Minnesota with 1 s TR and 1.6 mm resolution. The reprocessed 7T fMRI data released on April 10, 2018 was used in this study. A detailed description of the minimal preprocessing steps can be found in refs^2,19^ and of the FIX approach in ref.^20^.

The first dataset comprised of 3T rfMRI data was acquired for 355 subjects, including 143 males and 212 females. The second dataset was comprised of 7T rfMRI data for 184 subjects, including 72 males and 112 females. The third dataset was comprised of 3T task fMRI (tfMRI) data for seven task domains (Emotion, Gambling, Language, Motor, Relational, Social and Working Memory) for the same 355 subjects considered in the first dataset. Note that the 355 subjects selected for this study were the same ones as those considered in ref.^14^ and all the subjects who were scanned with the 7T imaging scanner. However, 12 subjects (5 males and 7 females) from the first dataset, 10 subjects (2 males and 8 females) from the second dataset, and 34 subjects (12 males and 22 females) from the third dataset were removed from further analysis in this study for two reasons. First, HCP data was missing an fMRI session. Second, head motion artefact occurred, which was detected when the average framewise displacement exceeding four standard deviations^21^.

The first dataset with 3T rfMRI data provided a baseline for studying intrinsic individual connectivity patterns for subject and sex identification. The second dataset with 7T rfMRI data, on the other hand, was chosen to study medium-term changes in the intrinsic individual connectivity, if any, because data was acquired about 6 to 12 months after subjects were scanned with the 3T imaging scanner. The third dataset was chosen to study intrinsic individual connectivity patterns obtained using the 3T rfMRI data in the 3T tfMRI data.

The 3T rfMRI data was acquired in four runs of approximately 15 minutes each, two runs with right-to-left (RL) and left-to-right (LR) phase encoding protocols in the first session on day 1 and two runs in the second session on day 2. However, the 7T rfMRI data was collected with phase encoding in posterior-anterior (PA) and anterior-posterior (AP) directions in four runs in two sessions on two consecutive days, similar to the acquisition of the 3T rfMRI data. In all cases in this study, the functional connectivity data from the two runs with different phase encoding protocols were averaged.

Parcellation was performed using an atlas with 90 brain functional regions of interest (fROIs) or nodes defined across 14 major RSNs (https://findlab.stanford.edu/functional_ROIs.html). Time series of the fROIs obtained using FSL [FSL-fslmeants]^22–24^ were detrended and demeaned [MATLAB-detrend], and the rfMRI data was bandpass filtered [MATLAB-filtfilt] in the range of 0.01634 Hz–0.15 Hz to improve identification of the resting-state fluctuations^21,25,26^ (see Fig. S1 in Supplementary Information for a schematic of the main data processing steps followed for subject identification in this study. Also, for clarity and completeness, the software package and commands, with any options, used to obtain the results are included within square brackets throughout the manuscript).

### Functional connectivity

**Figure 1:**
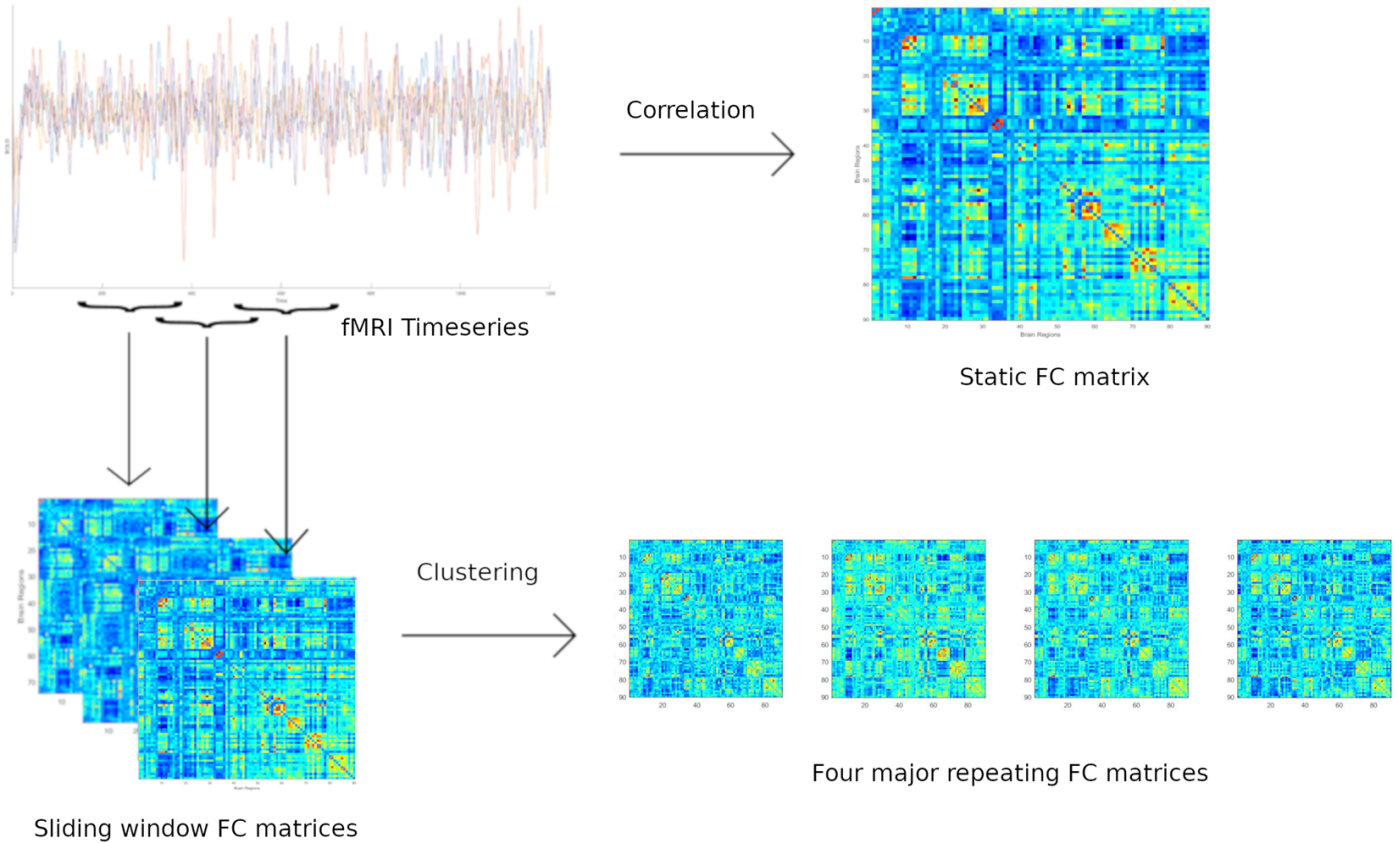
Static and dynamic FC matrices derived from fMRI time series. Static FC was calculated using Pearson correlation coefficients of the entire time series; however, dynamic FC was calculated considering a moving window of the time series and finding the major repeating FC matrices using a clustering algorithm.

Functional connectivity matrices for all the subjects in the three datasets were obtained using three methods: (i) Pearson sFC - Pearson correlation coefficients [MATLAB-corr] followed by normalization to z scores using the Fisher transformation; (ii) Partial sFC - partial correlation coefficients using the inverse covariance^27^ [MATLAB-inv, cov] followed by normalization to z scores using the Fisher transformation; and (iii) Pearson dFC - Pearson correlation coefficients with a windowed analysis, which involved calculating the FC matrices using Pearson correlation coefficients for each window after z-score normalization [MATLAB-zscore] of time series^26^, followed by correlation coefficients normalization to z scores using the Fisher transformation and clustering the FC matrices using the K-means clustering algorithm^7^ [MATLAB-kmeans] to extract the four most repeated states^6^ and arrange them in their decreasing order of occurrence [MATLAB-sort]. A window length of 85 TR (equivalent to 61.2 s, which was 1/fmin, where fmin was the minimum frequency of the bandpass filter) and a step size of 5 TR (equivalent to 3.6 s) were used for the 3T images. For the 7T images, a window length of 60 TR (60 s) was used with a step size of 5 TR (equivalent to 5 s). Figure 1 illustrates the sFC and dFC methods for generating the FC matrices. The sFC methods (i.e., Pearson sFC and Partial sFC) yielded 90 × 90 symmetric matrices, while the dFC method created four 90 × 90 symmetric matrices; therefore, only the upper or lower triangular part of these FC matrices needed to be considered during analysis.

### Similarity measure

Similarity between two FC matrices was determined using the cosine similarity measure. If *X*_*i*_ and *X*_*j*_ are two vectors formed by concatenating the *n* (*n* − 1)/2 upper triangular elements of the (*n* × *n*) functional connectivity matrices *FC*_*i*_ and *FC*_*j*_, respectively, then the cosine similarity measure between the two FC matrices is given by the inner product of the two vectors divided by their norms [MATLAB-pdist]:

1 $$C{S}_{i,j}=\frac{{X}_{i}\cdot {X}_{j}}{||{X}_{i}||\,||{X}_{j}||}$$

Values of the cosine similarity measure vary from −1 to 1, indicating maximal dissimilarity to maximal similarity. The cosine similarity measure was used in this study because of its simplicity and it was shown to provide better distinction compared to the Pearson correlation coefficient^6^. No effort was made in this study to compare various similarity measures and select one to satisfy some criterion for matching FC matrices.

### Edge analysis

Three quantitative measures were used to understand the ability of the three FC methods to identify each subject and their sex. First, edge consistency^11^, defined as those edges whose strengths were almost the same among all subjects, was used to identify edges that were consistent among all subjects. Therefore, high consistency edges do not contribute significantly to identifying a subject from other subjects. Edge consistency was determined by first calculating the standard deviation [MATLAB-std] of the edge strengths for all the subjects and then using a percentile [MATLAB-prctile] threshold to select those edges with a low standard deviation. Second, edge variability, defined as those edges that were not consistent among all subjects, on the other hand was used to identify edges that vary among all subjects. Therefore, high variability edges contribute significantly to identifying a subject from other subjects. Edge variability was calculated similar to edge consistency except for using a percentile threshold to select those edges with a high standard deviation. Finally, a modified form of the differential power of an edge^11^ was used to identify edges unique to each subject across different sessions and different from other subjects irrespective of the session when their data was acquired. The logarithmic function was replaced by calculating the mean to avoid undefined values when the probability was zero. The modified differential power for edge *e* was calculated as:

2 $$DP(e)=\frac{1}{N}({\sum }_{i=1}^{N}\,{P}_{i}(e))$$

where

3 $${P}_{i}(e)=(|{\varphi }_{ii} > {\varphi }_{ij}|+|{\varphi }_{ii} > {\varphi }_{ji}|)/2(N-1),\,i\ne j$$

4 $${\varphi }_{ij}(e)=\frac{{X}_{i}^{S1}(e)\ast {X}_{j}^{S2}(e)}{||{X}_{i}^{S1}||\,||{X}_{j}^{S2}||}$$

here $|{\varphi }_{ii} > {\varphi }_{ij}|$ means that the probability of *ϕ*_*ii*_ within the same subject is greater than *ϕ*_*ij*_ between two different subjects, ${X}_{i}^{Sk}$ is the vector formed by concatenating the *n*(*n* − 1)/2 upper triangular elements of the functional connectivity matrix *FC*_*i*_ for session *Sk*, *n* represents the total number of nodes, *i* and *j* represent two different subjects for subject identification or males and females for sex identification, *N* is the total number of subjects in the dataset and *e* is the *eth* edge in the *FC*_*i*_ matrix. Edges with higher differential power therefore contribute more to actually distinguish a subject from other subjects and to identify their sex.

### Subject identification using 3T and 7T rfMRI data

From the pool of 343 subjects in the first dataset obtained with the 3T imaging scanner, FC data from one of the two sessions for each subject was randomly selected to establish the known baseline. The remaining 343 rfMRI sessions were considered as unknown targets, which were then matched with their corresponding baseline to identify the subjects uniquely. Note that for the Pearson dFC method, the four states obtained using the K-means clustering algorithm were employed as the baseline for comparison with the four states obtained for the target subject. The cosine similarity was calculated between each baseline (4005 × 1 vector representing edge strengths) and each target (4005 × 363 matrix with columns representing targets and rows representing edge strengths). In the next step, the highest cosine similarity value was identified [MATLAB-max]. Binary values were then assigned with a score of 1 when a subject was correctly identified and a score of 0 when the prediction was wrong. The success rate of each method was finally calculated as a percentage of correctly identified subjects. The aforementioned steps were repeated for 174 subjects in the second dataset with the 7T rfMRI data.

### Medium-term changes in intrinsic individual connectivity patterns

The first and second datasets were used to study how the intrinsic individual connectivity patterns may change over a period of few months. One of the FC matrices from the two rfRMI sessions for 164 subjects from the 7T imaging scanner was randomly selected as the baseline. The 328 FC matrices from the two 3T rfRMI sessions for these subjects were selected as the target FC matrices. Again, each target subject was uniquely matched with its baseline based on the largest cosine similarity value and the percentages of correctly identified subjects were calculated.

### Subject identification using 3T rfMRI and 3T tfMRI data

Baseline FC matrices using the 3T rfMRI data from the first dataset were created as previously noted. However, the target FC matrices were for each of the seven task domains. Functional connectivity data for 321 subjects for each task domain were matched with their resting-state FC based on the largest cosine similarity value, and the percentages of correctly identified subjects were calculated as previously noted.

### Sex identification using 3T rfMRI data

Sex identification was performed to compare the performance of the three methods for group-level analysis. The leave-one-out strategy was used to create FC group averages removing the FC data of the subject to be predicted. The FC group averages for male and female subjects were calculated by randomly selecting one of the two sessions for each subject. Then each selected session for each of the 343 subjects (138 males and 205 females in the first dataset) was compared with the group averages of male and female FC matrices using the cosine similarity value. The predicted sex was then compared to the actual sex of the subject to calculate the accuracy percentage in sex identification.

## Results

### Subject identification using 3T and 7T rfMRI data

Table 1 shows the mean accuracy percentages for identifying subjects and the number of maximum subjects misidentified for 1,000 program runs. The partial sFC method was clearly able to correctly identify every subject in the 3T-3T scenario, whereas Pearson dFC and Pearson sFC methods misidentified a maximum of 2 and 40 subjects, respectively. For the 7T-7T scenario, the partial sFC method again had the highest accuracy with a maximum of 1 subject misidentified, compared to a maximum of 8 and 25 misidentified by the Pearson dFC and Pearson sFC methods, respectively. The subject misidentified by partial sFC was the same individual over trials, and this individual was also misidentified across methods. However, different subjects were misidentified across the other two methods. The 7T-3T scenario is the most challenging for two reasons. First, the FC matrices were for data acquired several months apart. Second, data was acquired on two different scanners with different magnetic field strengths. Because this study focused on the successful identification using intrinsic individual connectivity patterns, differences in scanners and acquisition parameters were not of interest. The partial sFC method once again performed best and was able to identify almost all the subjects with a maximum of 3 misidentified compared to 18 and 66 by the Pearson dFC and Pearson sFC methods, respectively. The Pearson dFC method was able to better capture the intrinsic individual connectivity patterns compared to Pearson sFC, but was not able to outperform the partial sFC method. The mean cosine similarity values between the FC matrices for the various identification cases are listed in Supplemental Table S1.

**Table 1:** Mean subject identification accuracy percentages and maximum number of misidentifications for 1000 program runs.

Baseline Subject-Target Subject	Pearson sFC		Partial sFC		Pearson dFC	
	Mean Accuracy Percentage	Maximum Number of Misidentifications	Mean Accuracy Percentage	Maximum Number of Misidentifications	Mean Accuracy Percentage	Maximum Number of Misidentifications
3T-3T	91.16	40	100.00	0	99.56	2
7T-7T	89.26	25	99.71	1	97.58	8
7T-3T	83.92	66	99.62	3	96.74	18

### Edge analysis in subject identification

**Figure 2:**
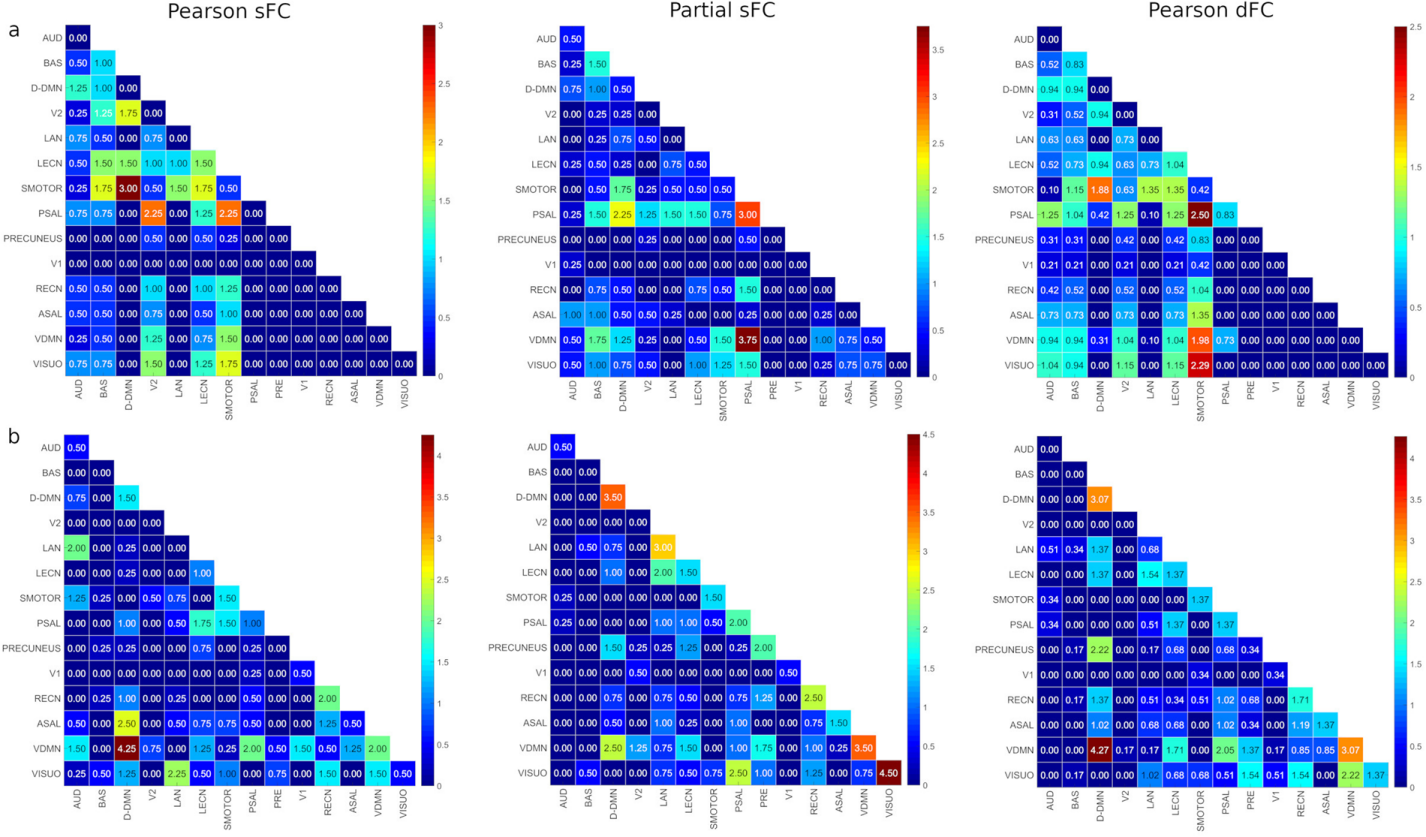
(**a**) Edge consistency percentages within and between RSNs for Pearson sFC, partial sFC and Pearson dFC methods at 5 percentile threshold. The color bar to the right of each figure displays the mapping of edge consistency percentage values to the color map. (**b**) Edge variability percentages within and between RSNs for Pearson sFC, partial sFC and Pearson dFC methods at 95 percentile threshold. The color bar to the right of each figure displays the mapping of edge variability percentage values to the color map. AUD-Auditory, BAS-Basal Ganglia, DDMN-Dorsal Default Mode Network, V2-Primary Visual, LAN-Language, LECN-Left Executive Control Network, SMOTOR-Sensorimotor, PSAL-Posterior Salience, PRE-Precuneus, V1-High Visual, RECN-Right Executive Control Network, ASAL-Anterior Salience, VDMN-Ventral Default Mode Network, VISUO-Visuospatial.

Edge consistency and edge variability were calculated using one standard deviation together with 5 percentile and 95 percentile thresholds, respectively. Figure 2 shows the percentages of the number of edges within and between RSNs and Table 2 summarizes the contribution percentage of each RSN toward edge consistency and edge variability. Except for the BAS network, which contains thalamus, caudate and frontal gyrus regions, the three methods identified different major contributing networks for edge consistency, and DDMN, VDMN and VISUO were common among all three methods for edge variability.

**Table 2:** Edge consistency (5 percentile threshold) and edge variability (95 percentile threshold) percentages for RSNs (top four contributing RSNs are shown in bold font).

RSN	Edge Consistency Percentage			Edge Variability Percentage		
	Pearson sFC	Partial sFC	Pearson dFC	Pearson sFC	Partial sFC	Pearson dFC
AUD	6.25	4.25	7.92	6.75	1.00	1.19
BAS	**10.50**	**10.25**	**10.00**	1.25	1.00	0.85
DDMN	8.50	**10.50**	6.35	**13.00**	**10.50**	**14.68**
V2	**12.75**	4.00	9.06	1.25	2.00	0.17
LAN	4.50	4.50	4.27	6.50	**10.75**	7.51
LECN	**14.00**	6.50	**11.25**	6.25	9.50	9.73
SMOTOR	**17.25**	8.00	**17.29**	7.75	3.00	3.24
PSAL	7.25	**19.50**	**9.38**	**9.25**	10.25	8.87
PRE	1.25	0.75	2.29	2.75	9.50	8.19
V1	0.00	0.25	1.25	2.25	1.00	1.37
RECN	4.25	5.50	3.02	7.25	9.50	**9.90**
ASAL	3.25	5.25	4.27	8.50	5.25	7.17
VDMN	4.25	**12.50**	7.08	**17.25**	**14.25**	**16.89**
VISUO	6.00	8.25	6.56	**10.00**	**12.50**	**10.24**

AUD-Auditory, BAS-Basal Ganglia, DDMN-Dorsal Default Mode Network, V2-Primary Visual, LAN-Language, LECN-Left Executive Control Network, SMOTOR-Sensorimotor, PSAL-Posterior Salience, PRE-Precuneus, V1-High Visual, RECN-Right Executive Control Network, ASAL-Anterior Salience, VDMN-Ventral Default Mode Network, VISUO-Visuospatial.

**Figure 3:**
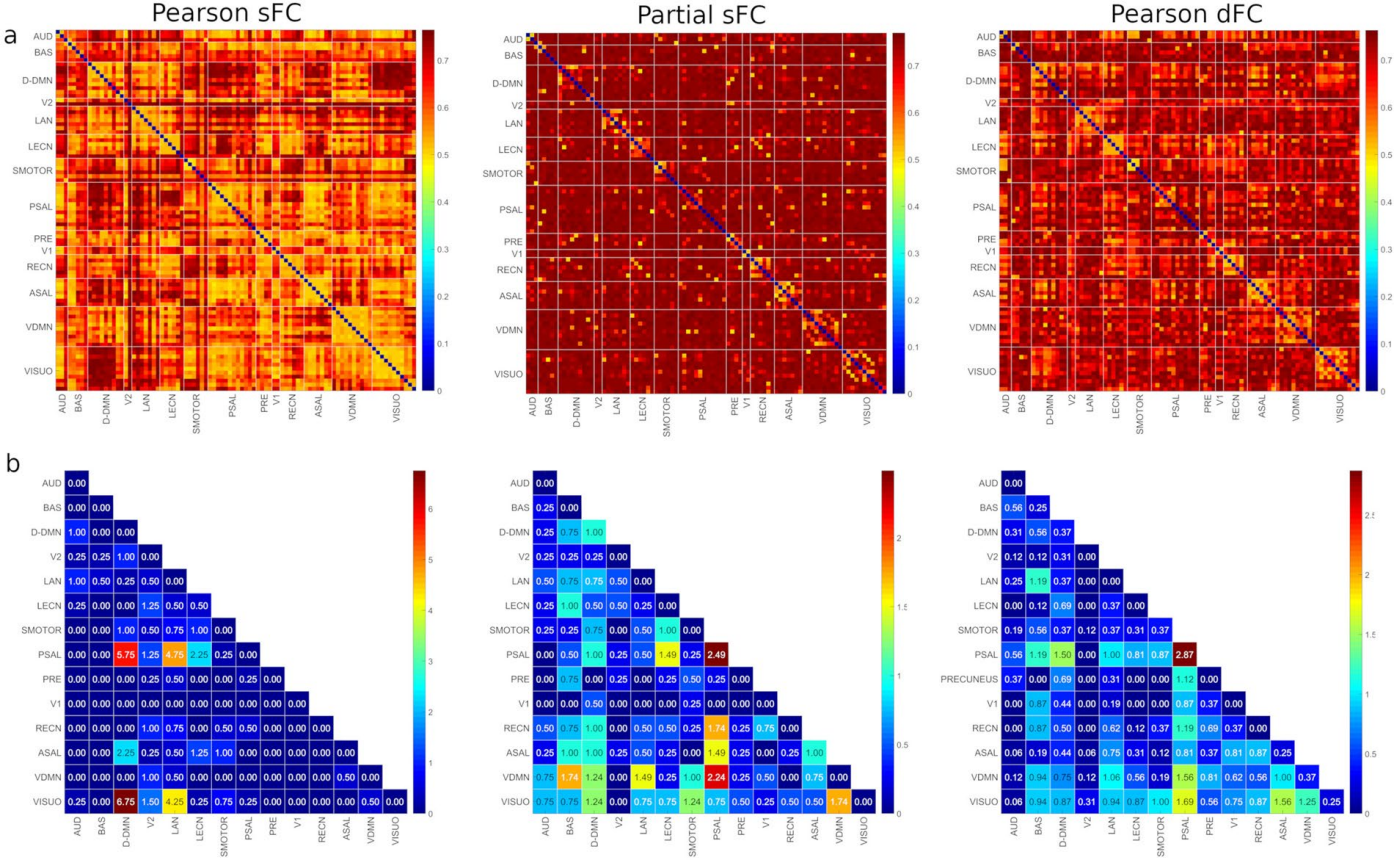
(**a**) Differential power of edges using Pearson sFC, partial sFC and Pearson dFC methods. The color bar to the right of each figure displays the mapping of edge differential power values to the color map. (**b**) Edge contribution percentages in subject identification at 95 percentile threshold. The color bar to the right of each figure displays the mapping of edge contribution percentage values to the color map. AUD-Auditory, BAS-Basal Ganglia, DDMN-Dorsal Default Mode Network, V2-Primary Visual, LAN-Language, LECN-Left Executive Control Network, SMOTOR-Sensorimotor, PSAL-Posterior Salience, PRE-Precuneus, V1-High Visual, RECN-Right Executive Control Network, ASAL-Anterior Salience, VDMN-Ventral Default Mode Network, VISUO-Visuospatial.

The higher warm zones in Fig. 3a show that many of the edges determined using the partial sFC method have a higher differential power compared to the Pearson dFC method, which in turn had higher values than Pearson sFC corroborating the subject identification accuracy results shown in Table 1. To identify the major RSNs with the highest differential power, a 95 percentile threshold was applied and the results obtained are shown in Fig. 3b. The number of active edges in an RSN was used to calculate the contribution of each RSN. Table 3 summarizes the contribution percentages of all the RSNs calculated using the ratio of number of edges in a network to total number of edges active after thresholding. As expected, the RSNs with higher differential power, which include DDMN, PSAL, VDMN and VISUO, also have higher edge variability (Table 2).

**Table 3:** RSN contribution percentages based on differential power at 95% threshold in subject identification (top four contributing networks highlighted in bold font).

RSN	Pearson sFC	Partial sFC	Pearson dFC
AUD	2.75	3.98	2.62
BAS	0.75	5.97	7.12
DDMN	**18.00**	**12.93**	**9.43**
V2	9.00	2.48	1.19
LAN	**14.75**	6.96	7.43
LECN	7.25	6.96	4.18
SMOTOR	5.75	6.21	4.87
PSAL	**15.25**	**12.93**	**16.04**
PRE	1.00	3.23	5.31
V1	0.00	2.23	5.31
RECN	2.75	6.96	7.05
ASAL	5.75	7.46	7.62
VDMN	2.50	**11.94**	**9.93**
VISUO	**14.5**	**9.70**	**11.92**

AUD-Auditory, BAS-Basal Ganglia, DDMN-Dorsal Default Mode Network, V2-Primary Visual, LAN-Language, LECN-Left Executive Control Network, SMOTOR-Sensorimotor, PSAL-Posterior Salience, PRE-Precuneus, V1-High Visual, RECN-Right Executive Control Network, ASAL-Anterior Salience, VDMN-Ventral Default Mode Network, VISUO-Visuospatial.

### Subject identification using 3T tfMRI data

Table 4 shows the mean accuracy value and the number of maximum subjects misidentified for 1,000 program runs for identifying subjects with one of the two 3T rfMRI sessions randomly selected as the baseline and the seven tfMRI data as the target, one at a time. Except for the emotion task, the partial sFC method had the best accuracy, followed by the Pearson dFC method. On the other hand, the accuracy of the Pearson sFC method varied from 7% to 21.3%. These mean accuracy percentages are very low and unsatisfactory. The mean cosine similarity values between the FC matrices for the various identification cases are listed in Supplemental Table S2.

**Table 4:** Comparison of 3T rfMRI (baseline) and 3T tfMRI (target) mean subject identification accuracy percentages.

Task	Pearson sFC		Partial sFC		Pearson dFC	
	Mean Accuracy Percentage	Maximum Number of Misidentifications	Mean Accuracy Percentage	Maximum Number of Misidentifications	Mean Accuracy Percentage	Maximum Number of Misidentifications
Emotion	21.30	266	70.52	114	71.59	108
Gambling	9.34	302	83.33	70	52.63	179
Language	10.16	300	93.97	27	65.97	135
Motor	8.90	302	86.48	55	62.03	142
Relational	10.09	299	73.97	102	53.14	176
Social	10.81	298	91.82	37	74.73	97
Working Memory	7.00	308	90.87	42	55.49	171

### Sex identification using 3T rfMRI data

Table 5 shows results obtained for sex identification for 1,000 program runs with the baseline obtained by randomly choosing one of the two sessions for each subject to calculate the group averages for male and female FC matrices, leaving out the FC matrix of subject to be predicted. The partial sFC method again outperformed the other two methods. The mean cosine similarity values between the subject and group FC matrices are listed in Supplemental Table S3.

**Table 5:** Mean sex identification accuracy percentages and maximum number of misidentifications for 1000 program runs.

Pearson sFC		Partial sFC		Pearson dFC	
Mean Accuracy Percentage	Maximum Number of Misidentifications	Mean Accuracy Percentage	Maximum Number of Misidentifications	Mean Accuracy Percentage	Maximum Number of Misidentifications
68.39	237	90.05	88	79.84	184

### Differential power of edges in sex classification

**Figure 4:**
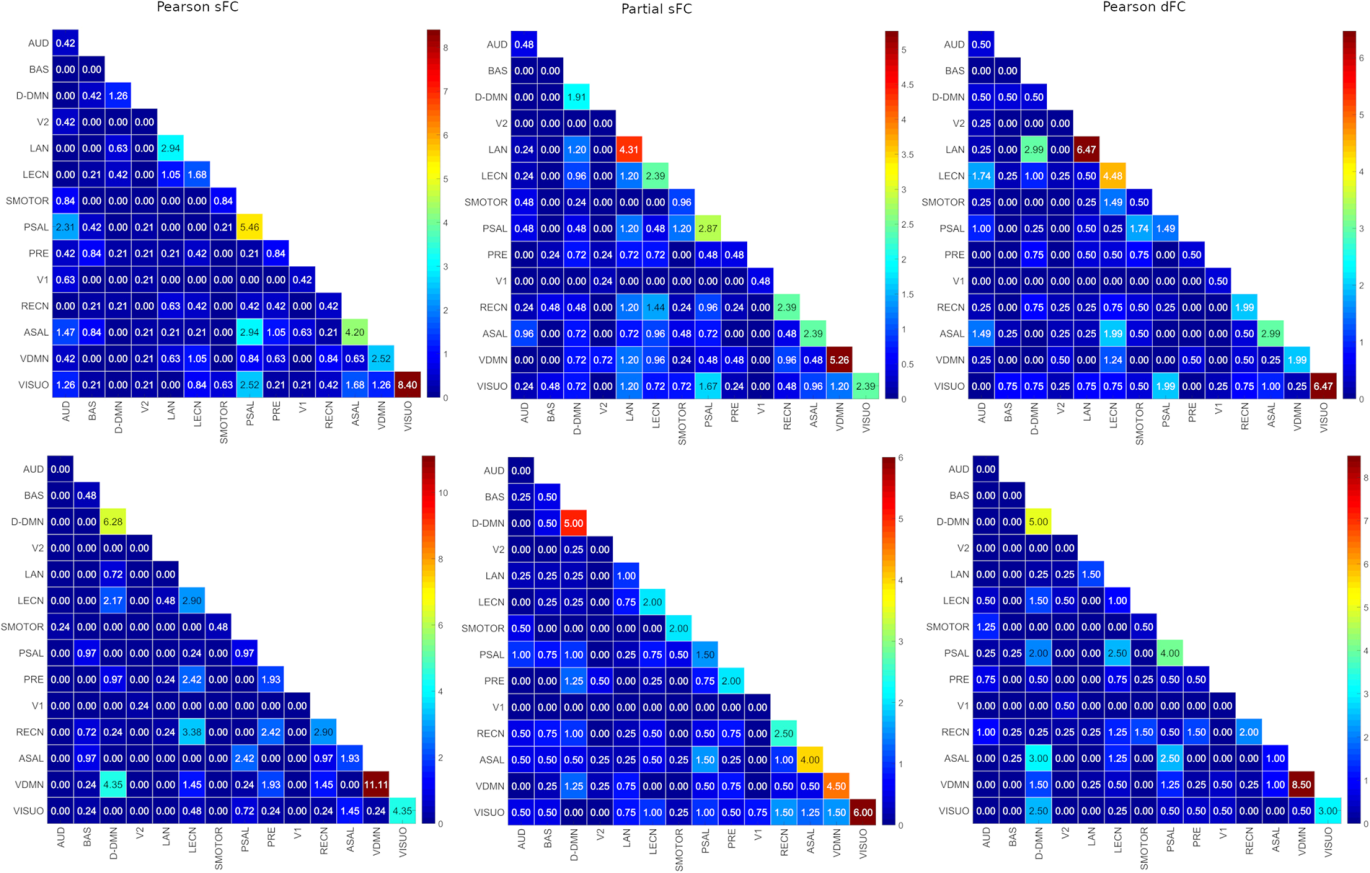
Differential power of edges using Pearson sFC, Partial sFC and Pearson dFC methods for males (TOP) and females (BOTTOM). The color bar to the right of each figure displays the mapping of edge differential power values to the color map. AUD-Auditory, BAS-Basal Ganglia, DDMN-Dorsal Default Mode Network, V2-Primary Visual, LAN-Language, LECN-Left Executive Control Network, SMOTOR-Sensorimotor, PSAL-Posterior Salience, PRE-Precuneus, V1-High Visual, RECN-Right Executive Control Network, ASAL-Anterior Salience, VDMN-Ventral Default Mode Network, VISUO-Visuospatial.

The edge percentages within and between RSNs contributing the most to differentiate between male and female subjects were found using a 95 percentile threshold and are shown in Fig. 4. Also, the edge percentages in each RSN are summarized in Table 6. Except for the DDMN appearing in all three methods for females, there was no RSN that was unique for sex identification.

**Table 6:** RSN contribution percentages in sex identification (top four contributing networks highlighted in bold font).

RSN	Pearson sFC		Partial sFC		Pearson dFC	
	Male	Female	Male	Female	Male	Female
AUD	8.19	0.24	3.35	3.5	6.47	3.75
BAS	3.15	3.62	1.20	4.50	1.74	0.75
DDMN	3.15	**14.73**	8.13	**11.25**	7.96	**16.5**
V2	1.68	0.24	1.20	1.25	1.74	1.50
LAN	6.30	1.69	**13.16**	4.75	**12.69**	2.50
LECN	6.30	**13.53**	10.05	6.25	**15.17**	**10.00**
SMOTOR	2.52	0.72	4.55	3.50	6.47	3.50
PSAL	**15.55**	5.56	**11.00**	10.00	7.46	**14.25**
PRE	5.67	10.14	4.55	7.00	3.48	5.50
V1	2.10	0.24	0.72	0.75	0.75	1.50
RECN	4.20	**12.56**	9.57	9.75	6.72	9.00
ASAL	**14.29**	7.73	8.85	**11.25**	**9.45**	9.00
VDMN	**9.03**	**21.01**	**12.68**	**10.75**	5.47	**14.5**
VISUO	**17.86**	7.97	**11.00**	**15.5**	**14.43**	7.75

AUD-Auditory, BAS-Basal Ganglia, DDMN-Dorsal Default Mode Network, V2-Primary Visual, LAN-Language, LECN-Left Executive Control Network, SMOTOR-Sensorimotor, PSAL-Posterior Salience, PRE-Precuneus, V1-High Visual, RECN-Right Executive Control Network, ASAL-Anterior Salience, VDMN-Ventral Default Mode Network, VISUO-Visuospatial.

## Discussion

The results presented here show that the intrinsic individual connectivity patterns can be used to identify subjects and their sex. A comparison was made between the commonly used sFC (Pearson correlation and partial correlation) and dFC (sliding window) measures. Results show that the intrinsic individual connectivity pattern was best captured by the partial sFC method, while the Pearson sFC method was not able to identify subjects accurately based on the intrinsic individual connectivity pattern. The dFC method performed better than the Pearson sFC method, but was not able to outperform the partial sFC method. The mean accuracy percentages with the Pearson sFC method was very low and unsatisfactory for identification purposes. A detailed analysis was performed to find the edges and networks that contributed to identifying the subjects. The top contributing RSNs varied among the three methods when edge consistency was considered, but there was commonality for edge variability. More importantly, the differential power results identified the networks that contributed the most to identifying the subjects and their sex. Findings from this study show that the heterogeneity in individual FC patterns can be exploited in future work to draw inferences about subjects in both health and disease.

The subject identification results, while complementing previous studies using the Pearson sFC^11^ and Pearson dFC^12^ methods, advocate the use of the partial sFC method for brain network analysis. Because of the use of different parcellation schemes, which has been shown to yield different results^2,11^, it is not possible to perform a fair comparison of the accuracy results obtained in this study with those obtained by others. The repeating states from the dFC matrices were found to have significant differences for some edge strength values, which enabled the Pearson dFC method to outperform the Pearson sFC method. To truly assess whether dFC would outperform sFC, a fourth comparison would have been necessary, that is, partial dFC. However, since the number of nodes that can be included in a partial correlation analysis is limited when the time series are short, the comparison would not have been valid. This is because of a reduction in the temporal degrees of freedom when nodes are regressed out to calculate an edge^28^. If the inverse of the covariance matrix is used to calculate the partial correlations, then the computational burden will be significant, and accurate results may not be reliably obtained^29^. The number of time series data points should be larger than the number of nodes to calculate the simple partial correlation, the accuracy of which improves, in most cases, as the time series length increases^30^. New methods are needed to accurately estimate the partial correlations without a huge computational load when the time series are short, which is typical in a windowed analysis, and more nodes are included. Thus, it remains unknown whether sFC outperforms dFC in general or whether when using partial correlation, dFC would actually be the preferred method. This is a topic for future investigations.

The results for edge consistency and edge variability corroborated with previous studies, and the performance of the three methods depended on what they identified as the major contributing networks. Ref. ^11^ reported the prefrontal cortex, motor, occipital and visual regions covered by the BAS, V2, SMOTOR and LECN networks as contributing at varying levels to consistency. Ref.^12^ showed the subcortical network covered by the BAS network to be the least variable among individuals, and ref.^31^ reported the SMOTOR, V1 and V2 networks to be the least variable among subjects. On the other hand, three networks (i.e., DDMN, VDMN and VISUO) were common among all three methods for edge variability. This result is consistent with ref.^31^, who showed that the frontoparietal control and attention networks had high functional variability followed by the default mode network with moderate variability.

Numerous studies have found that the DDMN and VDMN edges were active in individuals during rest^32,33^. In the case of the partial sFC method, the DDMN and VDMN networks appear as two of the top four contributing networks for both edge consistency and edge variability, which seems to be contradictory. However, it is not the case because the contribution of various edges in the two default mode networks are different, which reinforces the fact that consistency was not associated with local connectivity (within network) alone, but was also found in long range connectivity^31^. This makes it difficult to delineate some networks in functional connectivity analysis.

The high differential power of the DDMN, VDMN and VISUO networks is similar to the results obtained by ref.^11^. (frontoparietal and default mode network regions) and ref.^34^, who showed that the parietal cortex regions influenced individual differences. Ref.^31^. showed that the inter-subject variability was high in the frontoparietal control, ventral and dorsal attention regions followed by the default mode network. It should be pointed out that Table 3 shows that the VDMN network was having a higher differential power with the partial sFC and Pearson dFC methods, but the network contribution was small with the Pearson sFC method, which may be one of the reasons for its reduced subject identification accuracy.

Overall, subject identification using the tfMRI data showed reduced accuracy. This was expected as it is known that the connectivity patterns can be modulated by different task conditions, thereby reducing the identification accuracy^35–37^. Edge strengths in male and female FCs, which are known to have differences in structural^38,39^ and functional activation^2,40–42^, were better captured by the partial sFC method. The study by ref.^43^. showed that there were no significant differences among major RSNs in males and females, which can also be seen in the results as some RSNs contributed to distinguishing both males and females (LECN, VDMN, VISUO). The edge strengths within the RSNs rather than the RSNs themselves actually contributed to sex identification^2,41^.

Two factors that could potentially affect the results with a sliding window analysis are window length and the number of repeated states in the clustering algorithm. Various window lengths (65 TR–115 TR) and number of repeating states (4–8) were considered in this study. They all produced similar results for subject identification in the 3T-3T scenario, and no attempt was made to optimize these two parameters (see Supplemental Table S4). Edge analysis results were presented with a 95 percentile threshold. Likewise, similar results were obtained with different percentile thresholds for edge consistency, edge variability and differential power, and are included in Supplemental Tables S5–S7. No attempt was again made to optimize the threshold value, but can be easily done to explore the contribution of various networks for further functional connectivity analysis.

No consideration was given to family heritability in this study, but results presented here clearly show that the partial sFC method was superior for identifying intrinsic brain connectivity patterns compared to the Pearson sFC and Pearson dFC methods. The different imaging protocols (resolution and phase encoding) for the 7T and 3T rfMRI data should not be a concern because they have been shown not to affect connectome fingerprinting^44^.

The 7T-3T rfMRI results are intriguing because they show that the intrinsic individual connectivity patterns do not change in the medium term, and subject identification is still possible with images acquired several months apart. However, this is an area for further research and long-term longitudinal studies are needed to determine the influence of age or environmental factors on the intrinsic individual connectivity patterns. The results obtained clearly show that it is possible to not only uniquely identify a subject, but also to use the intrinsic brain connectivity pattern to identify the subject’s sex.

## Supplementary information

Supplementary Information

## Data Availability

The fMRI data used in this study can be downloaded from Human Connectome Project website (https://db.humanconnectome.org/). The 90 functional regions of interest parcellation atlas can be downloaded from the Stanford University Functional Imaging in Neuropsychiatric Disorders Lab website (https://findlab.stanford.edu/functional_ROIs.html). MATLAB scripts written to obtain the results are publicly available at github.com/sreevalsansmenon/IndividualBrainConnectivity.
