# Supplementary material for: A Comparison of Static and Dynamic Functional Connectivities for Identifying Subjects and Biological Sex Using Intrinsic Individual Brain Connectivity
Source: Sci Rep. 2019 Apr 5;9:5729. doi: 10.1038/s41598-019-42090-4 (PMC6450922; doi:10.1038/s41598-019-42090-4)
Supplement: Supplementary file 1 — Supplementary Information [file 41598_2019_42090_MOESM1_ESM.pdf]

A Comparison of Static and Dynamic Functional Connectivities for Identifying Subjects and Biological Sex Using Intrinsic Individual Brain Connectivity

Sreevalsan S. Menon, K. Krishnamurthy

Supplementary Information

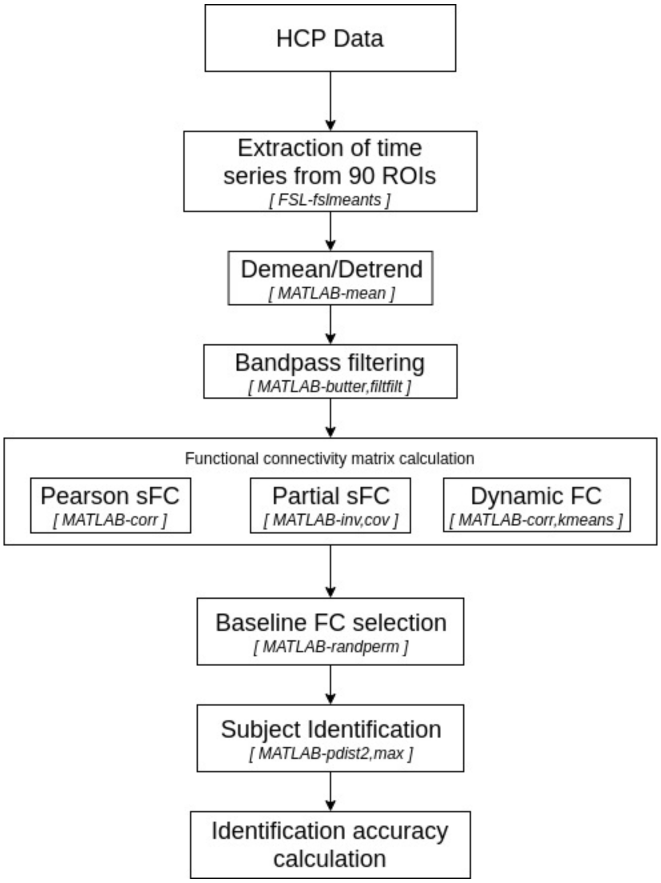

Figure S1: Schematic of main data processing steps for subject identification using rfMRI data. HCP data is available for download (https://db.humanconnectome.org). Results presented were obtained with FSL 5.0.11 and MATLAB 2018b software packages. Commands used in this study are shown in italic font within square brackets.

Table S1: Mean cosine similarity values between FC matrices during subject identification using 3T and 7T rfMRI data.

Data	Pearson sFC		Partial sFC		Pearson dFC	
	Intra-subject	Inter-subject	Intra-subject	Inter-subject	Intra-subject	Inter-subject
**3T-3T**	0.897	0.736	0.469	0.338	0.674	0.414
**7T-7T**	0.932	0.804	0.558	0.383	0.753	0.517
**7T-3T**	0.856	0.734	0.471	0.345	0.639	0.437

Table S2: Mean cosine similarity values between FC matrices during subject identification during subject identification using 3T rfMRI and 3T tfMRI data.

Task	Pearson sFC		Partial sFC		Pearson dFC	
	Intra-subject	Inter-subject	Intra-subject	Inter-subject	Intra-subject	Inter-subject
**Emotion**	0.768	0.682	0.266	0.202	0.447	0.354
**Gambling**	0.749	0.695	0.319	0.251	0.393	0.341
**Language**	0.741	0.688	0.366	0.283	0.400	0.300
**Motor**	0.761	0.694	0.340	0.264	0.405	0.342
**Relational**	0.723	0.658	0.307	0.234	0.382	0.307
**Social**	0.758	0.722	0.360	0.276	0.412	0.338
**Working Memory**	0.743	0.670	0.394	0.307	0.378	0.305

Table S3: Mean cosine similarity values between subject and group average FC matrices for sex identification using 3T rfMRI data. Intra-subject corresponds to male-male and female-female subjects and inter-subject corresponds to male-female and female-male subjects.

Pearson sFC		Partial sFC		Pearson dFC	
Intra-subject	Inter-subject	Intra-subject	Inter-subject	Intra-subject	Inter-subject
**0.865**	0.856	0.577	0.571	0.665	0.655

Table S4: Mean subject identification accuracy with different window size and number of repeating states for the Pearson dFC method with 3T fMRI data.

Window size (TR)	Number of repeating states				
	4	5	6	7	8
**65**	99.30	99.52	99.18	99.14	99.32
**75**	99.48	99.48	98.81	99.48	99.22
**85**	99.56	99.36	99.21	99.37	98.95
**95**	99.67	99.46	99.53	99.11	99.25
**115**	99.58	99.53	99.29	99.41	98.88

Table S5: Edge consistency percentages for networks at 10, 5 and 2.5 percentile thresholds (top four contributing networks highlighted in red color font). AUD-Auditory, BAS-Basal Ganglia, DDMN-Dorsal Default Mode Network, V2-Primary Visual, LAN-Language, LECN-Left Executive Control Network, SMOTOR-Sensorimotor, PSAL-Posterior Salience, PRE-Precuneus, V1-High Visual, RECN-Right Executive Control Network, ASAL-Anterior Salience, VDMN-Ventral Default Mode Network, VISUO-Visuospatial.

Network	Percentile Threshold								
	Pearson sFC			Partial sFC			Pearson dFC		
	10	5	2.5	10	5	2.5	10	5	2.5
**AUD**	7.23	6.25	6.50	4.11	4.25	4.00	7.56	7.92	6.38
**BAS**	10.85	10.50	8.00	10.22	10.25	12.50	9.54	10.00	9.57
**DDMN**	6.73	8.50	6.00	9.85	10.50	7.00	7.71	6.35	6.69
**V2**	8.85	12.75	16.00	3.49	4.00	2.50	7.25	9.06	12.16
**LAN**	4.74	4.50	5.50	4.74	4.50	4.00	4.43	4.27	4.71
**LECN**	11.85	14.00	13.50	7.11	6.50	7.50	9.31	11.25	12.61
**SMOTOR**	15.46	17.25	15.00	7.73	8.00	7.00	15.19	17.29	16.26
**PSAL**	10.35	7.25	8.50	18.70	19.50	21.00	14.50	9.38	8.36
**PRE**	2.24	1.25	0.00	0.75	0.75	1.00	2.06	2.29	2.28
**V1**	0.25	0.00	0.00	0.12	0.25	0.00	0.99	1.25	0.46
**RECN**	3.74	4.25	5.50	4.99	5.50	7.50	3.74	3.02	3.65
**ASAL**	4.61	3.25	5.50	6.36	5.25	6.50	3.28	4.27	3.34
**VDMN**	5.99	4.25	4.00	10.85	12.50	9.50	8.32	7.08	6.99
**VISUO**	7.11	6.00	6.00	10.97	8.25	10.00	6.11	6.56	6.53

Table S6: Edge variability percentages for networks at 90, 95 and 97.5 percentile thresholds (top four contributing networks highlighted in red color font). AUD-Auditory, BAS-Basal Ganglia, DDMN-Dorsal Default Mode Network, V2-Primary Visual, LAN-Language, LECN-Left Executive Control Network, SMOTOR-Sensorimotor, PSAL-Posterior Salience, PRE-Precuneus, V1-High Visual, RECN-Right Executive Control Network, ASAL-Anterior Salience, VDMN-Ventral Default Mode Network, VISUO-Visuospatial.

Network	Percentile Threshold								
	Pearson sFC			Partial sFC			Pearson dFC		
	90	95	97.5	90	95	97.5	90	95	97.5
**AUD**	4.74	6.75	7.00	1.25	1.00	1.50	1.71	1.19	0.68
**BAS**	2.00	1.25	1.00	1.25	1.00	1.00	1.44	0.85	0.68
**DDMN**	10.97	13.00	14.00	10.85	10.50	7.50	10.99	14.68	17.47
**V2**	1.87	1.25	1.00	2.37	2.00	3.00	0.81	0.17	0.00
**LAN**	8.98	6.50	7.50	10.22	10.75	10.00	8.74	7.51	4.79
**LECN**	6.98	6.25	6.00	9.73	9.50	8.00	8.74	9.73	8.90
**SMOTOR**	5.36	7.75	6.50	2.62	3.00	4.50	3.87	3.24	3.42
**PSAL**	10.10	9.25	9.00	10.10	10.25	9.00	9.01	8.87	9.59
**PRE**	4.61	2.75	4.00	8.60	9.50	10.00	6.76	8.19	8.22
**V1**	2.87	2.25	1.50	1.87	1.00	2.00	2.25	1.37	0.68
**RECN**	6.11	7.25	6.00	9.35	9.50	8.00	10.81	9.90	7.88
**ASAL**	6.61	8.50	7.50	5.36	5.25	4.50	8.02	7.17	6.85
**VDMN**	16.58	17.25	17.50	14.46	14.25	14.50	15.59	16.89	22.60
**VISUO**	12.22	10.00	11.50	11.97	12.50	16.50	11.26	10.24	8.22

Table S7: Network contribution percentages in subject identification at 90, 95 and 97.5 percentile thresholds for differential power (top four contributing networks highlighted in red color font). AUD-Auditory, BAS-Basal Ganglia, DDMN-Dorsal Default Mode Network, V2-Primary Visual, LAN-Language, LECN-Left Executive Control Network, SMOTOR-Sensorimotor, PSAL-Posterior Salience, PRE-Precuneus, V1-High Visual, RECN-Right Executive Control Network, ASAL-Anterior Salience, VDMN-Ventral Default Mode Network, VISUO-Visuospatial.

Network	Percentile Threshold								
	Pearson sFC			Partial sFC			Pearson dFC		
	90	95	97.5	90	95	97.5	90	95	97.5
**AUD**	2.87	2.75	1.50	3.62	3.98	4.50	3.15	2.62	2.49
**BAS**	1.12	0.75	0.50	6.23	5.97	9.50	8.08	7.12	8.60
**DDMN**	16.83	18	20.00	10.72	12.93	10.50	7.99	9.43	8.10
**V2**	9.73	9.00	8.50	2.00	2.48	2.00	1.19	1.19	1.00
**LAN**	12.97	14.75	18.00	7.61	6.96	6.00	8.08	7.43	8.35
**LECN**	6.73	7.25	4.50	7.11	6.96	5.50	4.37	4.18	3.87
**SMOTOR**	7.73	5.75	3.50	5.74	6.21	7.00	5.24	4.87	4.11
**PSAL**	12.97	15.25	16.00	13.59	12.93	11.50	15.61	16.04	17.33
**PRE**	1.00	1.00	0.50	3.87	3.23	4.50	5.68	5.31	5.36
**V1**	0.37	0.00	0.00	2.62	2.23	4.00	4.12	5.31	6.36
**RECN**	3.62	2.75	3.50	5.24	6.96	7.50	6.65	7.05	5.86
**ASAL**	7.23	5.75	5.00	7.11	7.46	6.00	6.80	7.62	7.36
**VDMN**	3.62	2.50	2.50	12.72	11.94	13.00	10.67	9.93	8.98
**VISUO**	13.22	14.50	16.00	11.85	9.70	8.50	12.36	11.92	12.22
